# NIH Disease Funding Levels and Burden of Disease

**DOI:** 10.1371/journal.pone.0016837

**Published:** 2011-02-24

**Authors:** Leslie A. Gillum, Christopher Gouveia, E. Ray Dorsey, Mark Pletcher, Colin D. Mathers, Charles E. McCulloch, S. Claiborne Johnston

**Affiliations:** 1 Department of Neurology, University of California San Francisco, San Francisco, California, United States of America; 2 Department of Epidemiology and Biostatistics, University of California San Francisco, San Francisco, California, United States of America; 3 Department of Neurology, Johns Hopkins University Medical Center, Baltimore, Maryland, United States of America; 4 Department of Health Statistics and Informatics, World Health Organization, Geneva, Switzerland; Yale University School of Medicine, United States of America

## Abstract

**Background:**

An analysis of NIH funding in 1996 found that the strongest predictor of funding, disability-adjusted life-years (DALYs), explained only 39% of the variance in funding. In 1998, Congress requested that the Institute of Medicine (IOM) evaluate priority-setting criteria for NIH funding; the IOM recommended greater consideration of disease burden. We examined whether the association between current burden and funding has changed since that time.

**Methods:**

We analyzed public data on 2006 NIH funding for 29 common conditions. Measures of US disease burden in 2004 were obtained from the World Health Organization's Global Burden of Disease study and national databases. We assessed the relationship between disease burden and NIH funding dollars in univariate and multivariable log-linear models that evaluated all measures of disease burden. Sensitivity analyses examined associations with future US burden, current and future measures of world disease burden, and a newly standardized NIH accounting method.

**Results:**

In univariate and multivariable analyses, disease-specific NIH funding levels increased with burden of disease measured in DALYs (p = 0.001), which accounted for 33% of funding level variation. No other factor predicted funding in multivariable models. Conditions receiving the most funding greater than expected based on disease burden were AIDS ($2474 M), diabetes mellitus ($390 M), and perinatal conditions ($297 M). Depression ($719 M), injuries ($691 M), and chronic obstructive pulmonary disease ($613 M) were the most underfunded. Results were similar using estimates of future US burden, current and future world disease burden, and alternate NIH accounting methods.

**Conclusions:**

Current levels of NIH disease-specific research funding correlate modestly with US disease burden, and correlation has not improved in the last decade.

## Introduction

The National Institutes of Health (NIH) is the largest public funder of biomedical research worldwide [1], [2], with a budget that has grown from $11.9 billion in 1996 to $28.5 billion in 2006 [3]. In the mid-1990s, Congress and the public raised concerns that disease-specific funding allocations by the NIH failed to adequately reflect burden of disease and incorporate public input [4]. In response, Congress requested that the Institute of Medicine (IOM) assess the NIH funding apportionment processes. In its 1998 report, *Scientific Opportunities and Public Needs: Improving Priority Setting and Public Input at the National Institutes of Health*, the IOM recommended improved tracking of disease-specific funding and development of a new priority-setting process [4].

A landmark study comparing disease burden to NIH funding levels was published in 1999 [5]. For 29 common conditions, the study examined a variety of measures of societal burden, recognizing that none by itself completely captures relative impacts of diseases. Disease incidence and prevalence were unrelated to funding, while mortality and years of life lost (YLLs) weakly correlated with funding. Disability-adjusted life years (DALYs)—a measure that estimates the equivalent number of healthy years lost due to disability or early death [6], [7]—were more strongly predictive. Using DALYs as the best single predictor, only 39% of the variance in NIH funding could be explained. The prior analysis was limited to evaluation of univariate predictors, and did not attempt to evaluate whether funding aligned with other measures of disease burden. The NIH Reform Act of 2006 re-emphasized the NIH's role in identifying research to meet public health challenges, and mandated submission of a biennial report to Congress regarding disease-specific funding amounts [8]. There has been no recent comprehensive study of US disease burden and NIH funding, and an analysis of only one of its institutes has been performed [9].

To determine whether the NIH has developed processes that better align funding with burden, we assessed the correlation between NIH funding and burden of disease, and compared results with those reported 10 years ago [5]. We also considered other potential predictors of funding and assessed the association of NIH funding with estimates of future and global disease burden.

## Methods

In a cross-sectional study, we compared measures of US and world disease burden and sociopolitical factors from 2004 to NIH funding levels in 2006. The study design was modeled on methods previously established [5], which used 1994 burden data and 1996 NIH funding to reflect an expected lag in availability of data on disease burden. Each disease was defined using pre-specified sets of *International Classification of Diseases, 9^th^ revision, Clinical Modification (ICD-9)* codes, which were applied to public sources of information on disease burden [10].

### Data Sources

Amounts of NIH funding for disease categories were obtained directly from the NIH for the year 2006 (Table 1). These estimates were annually consolidated from figures supplied by individual NIH Institutes and Centers (http://www.nih.gov/news/fundingresearchareas.htm) [11]. In 2006 and prior years, NIH Institutes and Centers categorized spending in a variety of manners to satisfy diverse reporting requirements, and calculated condition-specific total funds in a non-mutually exclusive manner. Thus, funding for a particular trial or biomarker could have been attributed to multiple conditions.

**Table 1:** NIH Research Funds and Measures of Disease Burden for 29 Conditions.

Condition or Disease	NIH Research Funds	Measure of Disease Burden in North America^*^ *thousands (rank)*				
	*millions of dollars (% of total)*	Incidence	Prevalence	Mortality	Years of Life Lost	Disability-Adjusted Life-Years
AIDS	2902 (24.3)	142 (19)	1275 (15)	14 (16)	279 (13)	583 (14)
Diabetes mellitus	1038 (8.7)	1200 (9)	21663 (2)	84 (7)	563 (7)	1473 (6)
Perinatal conditions	789 (6.6)	45 (21)	3516 (7)	17 (14)	578 (6)	793 (10)
Breast cancer	718 (6.0)	222 (13)	1875 (11)	53 (10)	488 (8)	684 (12)
Dementia	643 (5.4)	714 (10)	3108 (8)	132 (5)	306 (11)	1359 (8)
Alcohol abuse	511 (4.3)	2641 (6)	9553 (4)	8 (17)	121 (16)	1837 (4)
Dental and oral disorders	413 (3.5)	109774 (1)	41152 (1)	0	2 (27)	267 (18)
Cirrhosis	408 (3.4)	43 (22)	303 (21)	30 (12)	360 (10)	455 (16)
Ischemic heart disease	398 (3.3)	1336 (7)	2347 (10)	531 (1)	2695 (2)	3048 (3)
Schizophrenia	364 (3.1)	42 (23)	1561 (13)	1 (24)	5 (25)	522 (15)
Injuries	355 (3.0)	3747 (5)	241 (23)	182 (2)	3448 (1)	4484 (2)
Pneumonia	351 (3.0)	4178 (4)	75 (27)	68 (9)	294 (12)	315 (17)
Prostate cancer	348 (2.9)	149 (18)	1032 (16)	38 (11)	152 (14)	253 (20)
Stroke	342 (2.9)	373 (12)	2733 (9)	176 (4)	791 (4)	1336 (9)
Depression	335 (2.8)	16417 (3)	8207 (5)	1 (24)	3 (26)	4564 (1)
Asthma	283 (2.4)	1278 (8)	19100 (3)	4 (20)	53 (19)	755 (11)
Colorectal cancer	269 (2.3)	150 (17)	713 (18)	70 (8)	466 (9)	609 (13)
Lung cancer	266 (2.2)	196 (15)	706 (20)	181 (3)	1331 (3)	1384 (7)
Sexually transmitted diseases	264 (2.2)	Not available	Not available	0	2 (27)	65 (26)
Parkinson's disease	208 (1.7)	90 (20)	1025 (17)	21 (13)	66 (18)	263 (19)
Tuberculosis	150 (1.3)	11 (27)	11 (28)	1 (24)	7 (24)	10 (29)
Multiple sclerosis	110 (0.9)	9 (28)	176 (25)	4 (20)	45 (20)	118 (24)
Epilepsy	103 (0.9)	207 (14)	1677 (12)	2 (23)	31 (22)	160 (22)
Ovarian cancer	102 (0.9)	24 (26)	143 (26)	17 (14)	140 (15)	161 (21)
Cervical cancer	97 (0.8)	29 (24)	225 (24)	7 (18)	86 (17)	125 (23)
Chronic obstructive pulmonary disorder	67 (0.6)	429 (11)	6923 (6)	132 (5)	644 (5)	1647 (5)
Uterine cancer	28 (0.2)	25 (25)	301 (22)	7 (18)	45 (20)	84 (25)
Peptic ulcer disease	17 (0.1)	192 (16)	712 (19)	4 (20)	24 (23)	40 (27)
Otitis media	17 (0.1)	17679 (2)	1360 (14)	0	1 (29)	35 (28)

Funding rates by disease were obtained from the NIH for 2006. Estimates of incidence, prevalence, mortality, disability-adjusted life-years lost, and years-of-life-lost are total annual counts, in 1000s, for North America, obtained from the 2004 update of the World Health Organization's Global Burden of Disease project.

Data denoting disease burden were collected from multiple sources (Table 1). First, world and North American disease-specific data were obtained from the Global Burden of Disease (GBD) Project the World Health Organization (WHO) [12], [13], [14]. We used GBD's North American data for US estimates. The GBD systematically collects timely disease-specific epidemiologic information and models missing data to estimate a variety of measures of burden [15], [16]. We used 2004 GBD estimates because they were likely to be the most recent publicly available data at the time decisions were made for the 2006 funding cycle. No other centralized, systematic source of broad national disease-specific burden estimates was identified.

We evaluated five disease burden categories from the GBD: incidence, prevalence, mortality, YLLs, and DALYs [14]. YLLs for each disease were calculated by totaling the differences between life expectancy and age at death. DALYs were estimated based on YLL and on standardized weighting schemes for disability applied to those surviving with disease [6], [7]. GBD estimates for the US were based on analyses of comprehensive death certificate data, national incidence estimates, and a systematic review of published epidemiological studies.

To evaluate other estimates of disease burden, we used 2004 US inpatient and outpatient healthcare databases categorized by diagnosis, each of which includes large samples with weights to generate nationally representative estimates (Supporting Table S1). The number of hospital discharges, total length of stay, and mean hospital charges for disease-specific principal diagnoses were derived from the National Inpatient Sample (NIS) [17]. The number of visits to emergency departments and outpatient hospital clinics were derived from the National Hospital Ambulatory Care Survey (NHAMCS) and the National Ambulatory Care Survey (NAMCS) [18]. In both NIS and NHAMCS, community hospitals were defined as non-federal, short-stay hospitals [19], [20].

Public interest could also influence funding levels through lobbying efforts, additional funding support from private foundations, or by directly stimulating the interest of investigators, and this could influence funding levels. To begin to assess the influence of public interest, we determined the number of disease-specific newspaper articles published in the top 10 US newspapers with highest distribution, as well as broadcast television news reports from national networks, using disease-specific keyword searches of LexisNexis News [21] and Vanderbilt Television News Archive [22]. To estimate the influence of specific interest group advocacy, the total US disease-specific charity revenue was similarly calculated for public charities receiving more than $500,000 in public support [23].

Scientific productivity in a given area could stimulate further interest from researchers and the NIH, and could result in targeted funding. As surrogate measures of scientific productivity, we tallied the number of disease-specific patents submitted [24] and articles published and listed in PubMed [25] in the 10 medical journals with the highest impact factor scores using key word searches [26].

### Analysis

The relationship between 2006 NIH funding levels and 2004 US disease burden metrics was designated the *a priori* primary analysis. All predictor and outcome variables were log-transformed to reduce positive skew.

Univariate linear regression was first performed to replicate the prior study design [5]. This approach was preferred to correlation to allow for adjustment and to acknowledge that funding level was the dependent variable to be predicted. Expected funding levels in 2006 were calculated with correction for the log-transformation by applying measures of disease burden to fitted models predicting funding [27], [28]. To account for inflation, the actual and expected funding levels for 1996 were inflated to 2006 dollars [29]. Standard, forward stepwise multivariable linear regression analysis included all variables of disease burden, including those with p<0.05 in the final model. We did not evaluate interactions. F-statistics were used to estimate differences in explanatory power (significant if p<0.05). A separate forward stepwise multivariable model was constrained so that diseases with no burden would receive zero funding, mimicking a theoretical funding process that determines allocations proportional to the disease burden. For this model, we defined the dependent variable as the ratio of dollars to DALYs for each of the included conditions. Separate multivariable models included measures of public interest and scientific productivity. In additional sensitivity analyses, we evaluated the association of funding levels with worldwide disease burden in 2004, and with projections for 2015 and 2030 for both the US and worldwide [30]. A final analysis evaluated whether a new NIH accounting method for disease-specific funding introduced in 2007 produced different results. Explanatory power of the models was estimated with adjusted R^2^ values; an adjusted R^2^ value was also determined for the analysis of 1996 funding levels using data derived from this prior publication. The Stata statistical package (Version 10, College Station, Texas) was used for all analyses.

## Results

In 2006, the total NIH budget was $28.5 billion, with $11.9 billion devoted to the 29 included conditions. Disease funding ranged from $17 million (M) for peptic ulcer disease and otitis media, to $2902 M for AIDS, with a median of $335 M (±standard deviation $537 M; Table 1). Other metrics from the GBD (Table 1); US inpatient, emergency room, and outpatient (Supporting Table S1); and public interest and scientific opportunity (Supporting Table S2) varied by disease.

In the univariate analysis, NIH funding was most strongly associated with burden of disease measured in DALYs (p = 0.001; Table 2). YLLs (p = 0.03), inpatient hospital discharges (p = 0.05), and total hospital days (p = 0.02) were also associated with funding levels.

**Table 2:** Univariate Predictors of NIH Disease-Specific Funding in Fiscal Year 2006.

Predictor	Predicted change in funding associated with a 2-fold increase in the predictor*			
	Relative Increase	95% CI	*P*-Value	Adjusted R-squared Value
**Disease Burden Measure**				
Incidence	1.05	(0.91–1.20)	0.51	−0.02
Prevalence	1.15	(0.97–1.36)	0.10	0.06
Mortality	1.12	(0.98–1.27)	0.08	0.08
Disability-adjusted life-years	1.37	(1.16–1.63)	0.001	0.33
Years-of-life-lost	1.15	(1.01–1.31)	0.03	0.12
Number of hospital discharges	1.21	(1.00–1.46)	0.05	0.11
Total hospital days	1.24	(1.03–1.48)	0.02	0.15
Average hospital charges	1.49	(0.76–2.93)	0.23	0.02
Outpatient physician visits	1.11	(0.90–1.37)	0.30	0.004
Outpatient hospital and emergency room visits	1.10	(0.92–1.31)	0.27	0.01
**Public Interest Measure**				
Newspaper articles	1.13	(1.02–1.25)	0.02	0.14
Television news broadcasts	1.19	(1.04–1.36)	0.02	0.17
Charity revenue	1.07	(1.02–1.11)	0.004	0.24

*Predictors and outcome are log-transformed to reduce positive skew.

**Figure 1:**
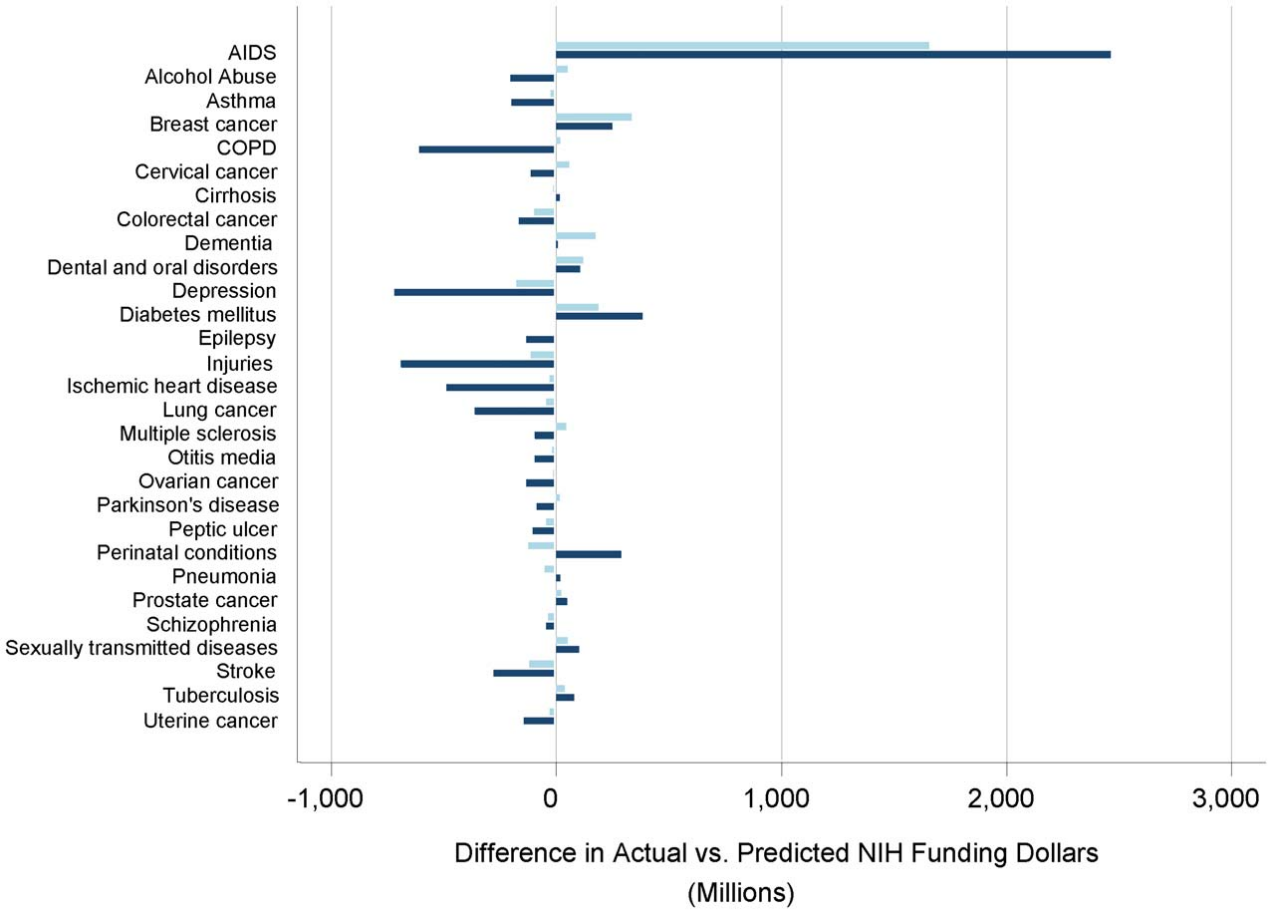
Ten-year Comparison of Differences Between Actual and Expected Disease-Specific NIH Funding Relative to US Burden of Disease in DALYs. A comparison of differences between actual and expected funding values as predicted by **DALYs burden alone in 1996** (**light blue**) and **2006** (**navy**). Negative values reflect actual funding dollars less than expected and positive values represent actual funding dollars more than expected.

In standard multivariable analysis, DALYs was the only significant predictor of NIH funding level retained in the final model, so the analysis became univariate. In 2006, the degree of correlation between NIH funding and disease burden as measured by DALYs alone was less than in 1996: Only 33% of NIH funding variance was explained in 2006 compared to 39% in 1996. Differences between actual and expected funding based on burden of disease in DALYs were estimated for 2006 and compared to 1996 funding levels (Table 3; Figure 1). Depression received the least funding compared to expected, and AIDS the most, consistent with findings from 1996. Relative to expected funding, AIDS, diabetes, and perinatal conditions were the three diseases with the largest amounts of funding, while depression, injuries, and COPD received the least funding (Table 3). The largest positive 10-year gains in actual NIH funding relative to expected were AIDS (+$809 M), perinatal conditions (+$420 M), and diabetes (+$193 M); by contrast, injuries (−$578 M), depression (−$541 M), chronic obstructive pulmonary disease (−$512 M), and ischemic heart disease (−$459 M) decreased most sharply (Figure 1).

**Table 3:** Ranked Differences between Expected and Actual NIH Funding According to Year of Funding and United States Disease Burden Measure(s) Used.

	Millions of Dollars*(ascending rank* * *)*			
	1996	2006
	Univariate†	Standard Univariate‡	Standard Multivariable with Public Interest Variables§	Constrained Multivariable¶
**Condition or Disease**				
Depression	−178 (1)	−719 (1)	−689 (2)	−951 (2)
Perinatal Conditions	−124 (2)	297 (27)	114 (23)	−194 (11)
Stroke	−121 (3)	−278 (6)	−288 (5)	−170 (13)
Injuries	−113 (4)	−691 (2)	−123 (12)	−721 (3)
Chronic obstructive pulmonary disorder	−101(5)	−613 (3)	−357(3)	−554(5)
Pneumonia	−52(6)	27 (21)	154 (27)	270 (27)
Peptic ulcer disease	−46(7)	−105 (14)	−75 (14)	−18 (19)
Lung cancer	−46(8)	−364 (5)	−328 (4)	−1358 (1)
Schizophrenia	−37(9)	−44 (18)	129 (24)	94 (24)
Ischemic heart disease	−30(10)	−490 (4)	−731 (1)	−721 (4)
Uterine cancer	−29(11)	−146 (10)	−94 (13)	−111 (15)
Asthma	−25(12)	−198 (8)	−235 (6)	40 (21)
Otitis media	−19(13)	−97 (15)	−70 (15)	−27 (18)
Colorectal cancer	−16(14)	−168 (9)	21 (19)	−519 (7)
Ovarian cancer	−15(15)	−135 (11)	−196 (9)	−337 (9)
Epilepsy	−10(16)	−133 (12)	−226 (7)	−101 (16)
Parkinson's disease	23(17)	−90 (17)	−183 (10)	−363 (8)
Cervical cancer	28(18)	−113 (13)	−44 (17)	−223 (10)
Prostate cancer	32 (19)	56 (22)	−3 (18)	68 (22)
Tuberculosis	44 (20)	89 (23)	96 (22)	97 (25)
Multiple sclerosis	52 (21)	−95 (16)	−222 (8)	−96 (17)
Sexually transmitted disease	58 (22)	110 (24)	153 (26)	198 (26)
Alcohol abuse	61 (23)	−202 (7)	152 (25)	−152 (14)
Cirrhosis	67 (24)	25 (20)	−55 (16)	−178 (12)
Dental and oral disorders	130 (25)	113 (25)	74 (20)	90 (23)
Dementia	183 (26)	18 (19)	−159 (11)	−524 (6)
Diabetes mellitus	197(27)	390 (28)	160 (28)	382 (28)
Breast cancer	346 (28)	258 (26)	92 (21)	39 (20)
AIDS	1664 (29)	2474 (29)	2306 (29)	1835 (29)

*Ascending rank, from most underfunded disease condition (indicated by negative numbers) to most overfunded (positive numbers) as predicted by each model.

† Univariate linear regression of the association between 2004 disease-specific Disability-Adjusted Life-Years (DALYs) and the outcome NIH dollars. Differences between expected and actual funding levels in 1996 are adjusted for inflation to 2006 dollar equivalents, but are otherwise unchanged from those reported by Gross *et al*.

‡ Standard univariate linear regression of the outcome NIH dollars as predicted by disease-specific DALYs. A stepwise forward multivariable model retained only DALYs as a predictor.

§ Standard multivariable linear regression of the outcome NIH dollars as predicted by disease-specific DALYs and charity revenue.

¶ The constrained model is the multivariable linear regression model where the predicted NIH dollars are obligated to be proportional to disease-specific DALYs after adjustment for total number of hospital discharges and average hospital charges.

In standard multivariable regression models including measures of public interest and scientific productivity, the total charity revenue for a given disease in 2006 (p = 0.04) was also predictive of funding in addition to DALYs (p = 0.006). A model including both variables explained 41% of the variation in NIH funding levels.

**Figure 2:**
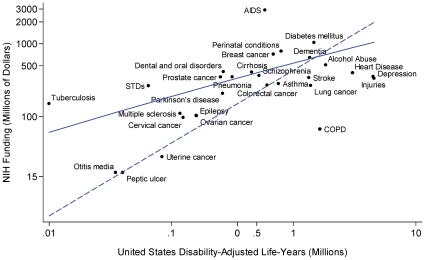
NIH Funding in 2006 and US Disease Burden in DALYs in 2004 for 29 Common Medical Conditions. The **solid line** represents the results of a traditional multivariable analysis, showing the relationship between US disease-specific DALYs burden and actual 2006 NIH funding dollars. The **dashed line** projects NIH funding levels in a similar multivariable model that requires that a disease with no burden receives no funding (constrained model). Though the models produce similar results, several diseases that would be considered overfunded in one model are considered underfunded in the other. For example, cervical cancer appears to be overfunded relative to the dashed line, while it is underfunded relative to the solid one.

**Figure 3:**
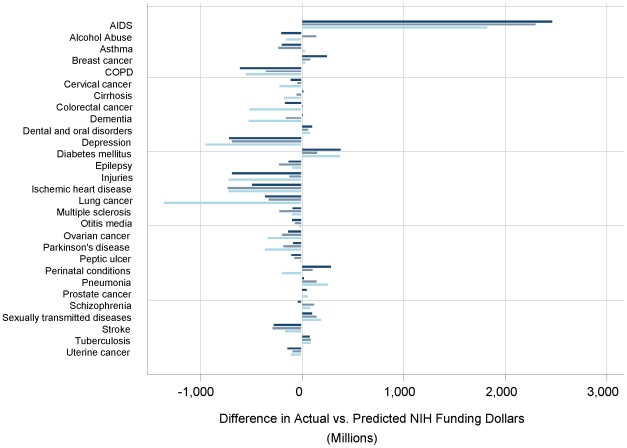
Differences Between Actual and Expected Disease-Specific Funding in 2006. Determinations of actual funding relative to expected funding were generally similar among separate analytic models predicting funding levels from disease burden measures. Univariate results are based on **DALYs alone** (**navy**), the only variable retained in a stepwise forward multivariable model. A traditional multivariable model including **public interest variables** (**grey-blue**) retained only DALYs and total charity revenue in the model. A **constrained multivariable** (**light blue**) model required an intercept of zero-zero to impose a requirement that conditions with no burden received no funding and retained DALYs, total number of US hospital discharges, and mean charge per hospitalization in 2004.

In multivariable models constrained to require that diseases resulting in no burden of illness receive no NIH funding (equivalent to requiring an intercept of zero-zero in the regression line, expressed in the dashed line of Figure 2), expected funding amounts were generally similar to those found with the unconstrained multivariable model (Table 3; Figure 3).

To determine if NIH funding might better correlate with world or future disease burden, we performed sensitivity analyses with global measures and future projections (2015 and 2030), all derived from the GBD project (Supporting Table S3). When restricted to global measures, mortality (p = 0.05) and DALYs (p = 0.001) were predictive of funding in univariate analyses (adjusted R^2^ values 0.11 and 0.30), but only DALYs were retained in all the multivariable models of both global measures and future predictions. Correlation of funding with disease burden was not improved when data utilizing new NIH accounting methods was used (adjusted R^2^ = 0.27) compared to prior methods (adjusted R^2^ = 0.34) applied to 2007 data, the first year for which the new methods were available.

## Discussion

In the 10 years since an initial assessment of the correlation of NIH funding with disease burden [5] and an IOM report recommending new NIH funding priority-setting criteria [4], NIH funding is no better aligned with US disease burden. Furthermore, diseases that were previously funded more than expected–such as AIDS, breast cancer, and diabetes–continue to receive funding greater than predicted by burden of disease, while most conditions that were previously underfunded remain underfunded. Adding measures of disease burden to the model and constraining it to assure that diseases with no burden would receive no funding minimally affected the overall relationship between burden and funding. Neither global nor future disease burden were more closely related to NIH funding, and newly implemented NIH disease-specific accounting practices did not improve the correlation.

Although the IOM report on NIH funding recommended ongoing assessment of alignment of NIH funding with disease burden, it also recognized other important criteria for setting funding priorities [4]. These criteria included research quality, scientific innovation and opportunity, portfolio diversification, and infrastructure building. Other experts have proposed a similar framework to guide funding decisions [31]. Additional factors to consider may include transmissibility or population risk, collateral benefits to disease control, and public interest. Given these other potential contributors to decision-making about disease funding, perfect alignment with DALYs or any other measure of disease burden would not be expected.

It is unclear why particular conditions remain under- or overfunded relative to disease burden. The difficulty of attributing basic science research—a large portion of the NIH portfolio—to individual diseases complicates implementation of any disease-based allocation process [32]; however, some discrepancies are particularly dramatic. Spending for AIDS research, the disease with greatest funding compared to expected, may be justified due to the potential threat associated with its spread, to past successes in treating and even eliminating other infectious diseases, and to a greater burden in lower income countries. However, AIDS funding remained greater than predicted even when worldwide and projected burden were considered, and strong political influences may be important in maintaining high levels of funding in the US. Also, congressionally-mandated research support for rare illnesses may explain greater funding for some diseases with little burden [33], [34], [35]. Conditions typically associated with substance use or mental health diagnoses tended to be underfunded (e.g., lung cancer, chronic obstructive pulmonary disease, alcoholism, and depression). Charity revenue, used as a proxy for disease-specific interest-group advocacy, was associated with funding levels and may have contributed through lobbying efforts or by providing collateral support for research and training to encourage NIH submissions in specific disease areas. The availability of proven cost-effective interventions (e.g., tobacco-related prevention strategies) [36] may also influence funding since development of new interventions may be unnecessary when effective strategies have already been identified [37].

Over the 10-year interval, funding for several conditions notably increased compared to expected. For example, the National Institute of Allergy and Infectious Diseases initiated a concerted response to bioterrorism following the terrorist attacks on Sept. 11, 2001, anthrax incidents, and severe acute respiratory syndrome (SARS) outbreak in 2002, and this may have augmented support for pneumonia-related research [38]. Conversely, the relative funding increase for perinatal conditions appears primarily due to a 55% reduction in associated DALYs over the last decade since funding increases over the same period paralleled overall growth in the NIH budget [39]. Finally, an increase in relative funding for diabetes research may have been precipitated by Congressional actions in 1997 requiring development of a comprehensive diabetes research plan and allocating $150 million to a new funding program for Type I Diabetes Research [40].

Lack of improvement in alignment between funding and disease burden may not indicate neglect of the 1998 IOM recommendations by the NIH; there are several other possible explanations. First, basic science research has consistently accounted for 55% of NIH spending and it is difficult to credit specific disease for much of this research, contributing uncertainty to the analysis and reducing correlations between funding levels and disease burden. Second, NIH funds committed at the time of the 1998 IOM publication could not be redistributed until their associated projects were completed, sometimes five or more years later; as a result, reallocations would be delayed for several years. Third, the distribution of funding among NIH Institutes is determined by Congress and incorporates input from NIH itself, scientists, health care providers, and special interest groups. Thus, fiscal and political constraints likely additionally tempered the NIH's ability to implement the IOM recommendations. Finally, without regard to overall disease funding alignment, substantial financing ($1.8 billion, 6.3% 2006 NIH budget) was invested in the creation of three new centers (National Institution for Biomedical Imaging and Bioengineering, Center for Complementary and Alternative Medicine, and National Center for Minority Health and Health Disparities) [41], establishment of the cross-cutting Roadmap Initiatives [42], and expanded emphasis on career training awards [43].

The NIH has recently taken steps to integrate more effectively the IOM priority-setting criteria. In 2007, the Division of Program Coordination, Planning, and Strategic Initiatives (DPCPSI) was established for the purposes of identifying scientific opportunities, public health challenges, and scientific knowledge gaps, and to improve portfolio analysis and priority-setting [44]. In 2009, a more consistent and transparent system for the tracking of disease-based funding was launched. Also, as part of the NIH's $10.4 billion allocation in the American Recovery & Reinvestment Act, $400 million will be dedicated to comparative effectiveness research that specifically evaluates the effects of clinical management on comprehensive public health outcomes such as mortality, morbidity, and quality of life [45].

Our study has several limitations. First, the accounting of disease funding in 2006 by NIH is not standardized nor is the reliability known [32]. However, no alternative source of information is publicly available and historical and new accounting methods yielded similarly poor correlations with burden. Second, we did not evaluate other sources of federal, nonprofit, and industry funding. Conditions well-funded by organizations other than the NIH may justify a corresponding decrease in NIH funding [46]. However, other sources of federal funding–The Centers for Disease Control and Prevention, Agency for Healthcare Research and Quality, and the Food and Drug Administration–distributed only 3.3% of all Department of Health and Human Services dollars dedicated to life sciences research in 2006. Similarly, the total life science research dollars spent by the Department of Defense and Department of Veterans Affairs was equivalent to less than 7% of the total amount disbursed by the NIH [47], [48]. Thus, the total dollars spent on complementary projects by other federal institutions does not fully explain the poor correlation between disease burden and NIH funding. Although biomedical and pharmaceutical industry research funding in the United States was 1.6 times the amount allocated by the NIH in 2006, fully 49% was dedicated to supporting clinical trials less likely to have a widespread public health benefit [49]. Unfortunately, funding by disease for these sectors is not available, except for limited therapeutic areas [50], and no such figures exist for private foundations. Third, global estimates of disease burden from the WHO GBD are uncertain due to incompleteness and bias, particularly in low-income countries [51]. However, these estimates are the best available and are particularly accurate for the US. Fourth, to permit comparisons to the prior study and to make the study feasible, many diseases and conditions funded by the NIH were omitted from our analysis. Still, an assessment of the responsive of NIH to prior recommendation was central to our study and power would not be expected to dramatically impact our findings. Fifth, the prior analysis utilized estimates for world market economies [5], while we used North American numbers. The data we used better approximates US burdens, strengthening our conclusions, but could affect the assessment of interval change. Since we found a poorer correlation with funding than previous estimates, this did not impact our conclusions. Finally, none of the measures of disease burden individually or collectively fully captures the health and economic cost of these conditions. A better metric might capture the true societal cost of disease through a comprehensive assessment of total healthcare costs and a valuation of both deaths and DALYs [52].

Overall, funding levels today are less well aligned with DALYs compared to 10 years ago, suggesting that the IOM's 1998 priority-setting recommendations have not been implemented effectively. Accounting for other measures of disease burden did not substantially improve alignment. As a recipient of substantial governmental support, clear articulation of the rationale for NIH spending may be expected by Congress and the public, and a lack of clear alignment with measures of public burden could encourage special interests to further erode the scientific independence of NIH or to raise questions about its management. The use of more consistent accounting methods for disease funding, more comprehensive measures of burden and future risk that include impact on health and expenditures, and the timely dissemination of benchmarks on the alignment of disease burden to funding could help to make NIH funding priorities more rationale and transparent.

## Supporting Information

Table S1 Additional Measures of Disease Burden for 29 Conditions.
(DOC)

Table S2 Public Interest and Other Measures for 29 Conditions.
(DOC)

Table S3 World and Future Disability-Adjusted Life-Years as Predictors of NIH Disease-Specific Funding in Fiscal Year 2006.
(DOC)
