# Supplementary material for: NIH Disease Funding Levels and Burden of Disease
Source: PLoS One. 2011 Feb 24;6(2):e16837. doi: 10.1371/journal.pone.0016837 (PMC3044706; doi:10.1371/journal.pone.0016837)
Supplement: Table S1 — Additional Measures of Disease Burden for 29 Conditions. (DOC) [file pone.0016837.s001.doc]

| **Table S1.** Additional Measures of Disease Burden for 29 Conditions. | | | | | |
| --- | --- | --- | --- | --- | --- |
| **Condition or Disease** | **Hospitalizations in the United States** | | | **Total Outpatient**  **Physician Visits**  *Thousands* | **Total Emergency and**  **Hospital Outpatient**  **Visits**  *Thousands* |
|  | **Number of**  **Discharges** | **Total Days of**  **Admission** | **Mean**  **Charges**  *Dollars* |  |  |
| AIDS | 73232 (19) | 673634 (17) | 41148 (3) | 1238 (22) | 249 (21) |
| Ischemic heart disease | 1934078 (2) | 7929720 (2) | 40165 (4) | 17886 (6) | 2668 (8) |
| Diabetes mellitus | 512318 (6) | 2715285 (7) | 20570 (16) | 45643 (2) | 6162 (2) |
| Perinatal conditions | 161358 (12) | 1710395 (9) | 39923 (5) | 1259 (21) | 255 (20) |
| Injuries | 2849668 (1) | 15103240 (1) | 28753 (9) | 55608 (1) | 37392 (1) |
| Breast cancer | 80287 (18) | 224804 (20) | 18369 (21) | 5891 (9) | 418 (14) |
| Dementia | 112331 (15) | 988513 (14) | 18677 (20) | 2480 (16) | 244 (22) |
| Alcohol abuse | 230174 (10) | 1035783 (13) | 9855 (28) | 1977 (18) | 1871 (10) |
| Dental and oral disorders | 45699 (22) | 146237 (24) | 16943 (24) | 3176 (15) | 2407 (9) |
| Cirrhosis | 111609 (16) | 714298 (16) | 32517 (8) | 585 (26) | 261 (19) |
| Pneumonia | 1417688 (3) | 7513746 (3) | 19383 (18) | 9664 (8) | 4397 (5) |
| Schizophrenia | 337816 (9) | 3952447 (5) | 32517 (19) | 2079 (17) | 893 (13) |
| Prostate cancer | 81234 (17) | 276196 (18) | 21329 (14) | 4122 (11) | 282 (17) |
| Stroke | 925076 (4) | 4717888 (4) | 24901 (11) | 4138 (10) | 1153 (11) |
| Depression | 474219 (7) | 3082424 (6) | 11115 (27) | 25601 (3) | 3595 (7) |
| Asthma | 418789 (8) | 1423883 (10) | 12096 (26) | 22307 (4) | 4570 (4) |
| Colorectal cancer | 155292 (13) | 1397628 (11) | 41499 (2) | 4029 (12) | 237 (23) |
| Lung cancer | 151755 (14) | 1183689 (12) | 35141 (7) | 3193 (14) | 303 (15) |
| Sexually transmitted diseases | 62059 (20) | 223412 (21) | 16159 (25) | 3750 (13) | 1096 (12) |
| Parkinson’s disease | 16236 (26) | 76309 (27) | 19748 (17) | 1367 (20) | 164 (25) |
| Tuberculosis | 8574 (29) | 134612 (25) | 48710 (1) | 543 (27) | 65 (26) |
| Multiple sclerosis | 20637 (25) | 105249 (26) | 17063 (23) | 1179 (23) | 164 (24) |
| Epilepsy | 57738 (21) | 259821 (19) | 20963 (15) | 900 (24) | 287 (16) |
| Ovarian cancer | 26354 (24) | 192384 (22) | 37017 (6) | 619 (25) | 65 (27) |
| Cervical cancer | 15048 (27) | 69221 (28) | 23602 (12) | 175 (29) | 28 (29) |
| Chronic obstructive  pulmonary disorder | 545618 (5) | 2673528 (8) | 17235 (22) | 22115 (5) | 4255 (6) |
| Uterine cancer | 39009 (23) | 171640 (23) | 23224 (13) | 356 (28) | 48 (28) |
| Otitis media | 9308 (28) | 26062 (29) | 7610 (29) | 17623 (7) | 4637 (3) |
| Peptic ulcer disease | 173315 (11) | 901238 (15) | 25072 (10) | 1481 (19) | 280 (18) |
