# Supplementary material for: NIH Disease Funding Levels and Burden of Disease
Source: PLoS One. 2011 Feb 24;6(2):e16837. doi: 10.1371/journal.pone.0016837 (PMC3044706; doi:10.1371/journal.pone.0016837)
Supplement: Table S2 — Public Interest and Other Measures for 29 Conditions. (DOC) [file pone.0016837.s002.doc]

| **Table S2.** Public Interest and Other Measures for 29 Conditions. | | | | | |
| --- | --- | --- | --- | --- | --- |
| **Condition or Disease** | **No. of**  **Newspaper**  **Articles** | **No. of**  **Television**  **News**  **Broadcasts** | **No. of**  **Patents**  **Submitted** | **No. of**  **Published**  **Journal Articles** | **Charity**  **Revenue**  *$ Thousands* |
| AIDS | 13424 (2) | 101 (1) | 157 (1) | 1124 (2) | 167015 (5) |
| Ischemic heart disease | 4205 (4) | 38 (4) | 18 (17) | 1216 (1) | 458559 (1) |
| Diabetes mellitus | 2134 (6) | 26 (6) | 31 (7) | 812 (6) | 456311 (2) |
| Perinatal conditions | 57 (24) | 0 (25) | 1 (24) | 246 (11) | 215881 (4) |
| Injuries | 16217 (1) | 57 (2) | 21 (13) | 1041 (4) | 0 (19) |
| Breast cancer | 1509 (8) | 44 (3) | 71 (3) | 540 (8) | 144519 (7) |
| Dementia | 2008 (7) | 21 (9) | 21 (13) | 178 (15) | 154226 (6) |
| Alcohol abuse | 1379 (9) | 4 (14) | 8 (19) | 204 (13) | 0 (19) |
| Pneumonia | 875 (11) | 2 (18) | 6 (20) | 477 (9) | 0 (19) |
| Dental and oral disorders | 0 (28) | 0 (25) | 0 (26) | 13 (28) | 1402 (16) |
| Cirrhosis | 34 (26) | 1 (21) | 5 (21) | 169 (16) | 9907 (10) |
| Schizophrenia | 332 (19) | 2 (18) | 27 (8) | 160 (17) | 0 (19) |
| Prostate cancer | 594 (16) | 12 (10) | 65 (4) | 180 (14) | 3537 (13) |
| Stroke | 7920 (3) | 35 (5) | 141 (2) | 1086 (3) | 2911 (15) |
| Depression | 3505 (5) | 22 (8) | 58 (5) | 827 (5) | 9291 (11) |
| Asthma | 902 (10) | 6 (12) | 51 (6) | 389 (10) | 3141 (14) |
| Colorectal cancer | 820 (12) | 5 (13) | 25 (11) | 139 (19) | 0 (19) |
| Lung cancer | 664 (13) | 10 (11) | 27 (8) | 235 (12) | 927 (17) |
| Sexually transmitted diseases | 605 (15) | 1 (21) | 3 (23) | 44 (27) | 0 (19) |
| Parkinson’s disease | 618 (14) | 1 (21) | 1 (24) | 124 (20) | 17684 (9) |
| Tuberculosis | 451 (18) | 4 (14) | 20 (15) | 555 (7) | 0 |
| Multiple sclerosis | 498 (17) | 2 (18) | 26 (10) | 123 (21) | 216559 (3) |
| Epilepsy | 294 (20) | 4 (14) | 11 (18) | 144 (18) | 22074 (8) |
| Ovarian cancer | 0 (28) | 1 (21) | 22 (12) | 99 (22) | 3714 (12) |
| Cervical cancer | 90 (22) | 4 (14) | 4 (22) | 86 (24) | 0 (19) |
| Chronic obstructive pulmonary disease | 247 (21) | 0 (25) | 19 (16) | 94 (23) | 2 (18) |
| Uterine cancer | 36 (25) | 0 (25) | 0 (26) | 7 (29) | 0 (19) |
| Otitis media | 75 (23) | 26 (6) | 0 (26) | 53 (26) | 0 (19) |
| Peptic ulcer disease | 23 (27) | 0 (25) | 0 (26) | 59 (25) | 0 (19) |
