# Supplementary material for: NIH Disease Funding Levels and Burden of Disease
Source: PLoS One. 2011 Feb 24;6(2):e16837. doi: 10.1371/journal.pone.0016837 (PMC3044706; doi:10.1371/journal.pone.0016837)
Supplement: Table S3 — World and Future Disability-Adjusted Life-Years as Predictors of NIH Disease-Specific Funding in Fiscal Year 2006. (DOC) [file pone.0016837.s003.doc]

| **Table S3.** World and Future Disability-Adjusted Life-Years as Predictors of NIH Disease-  Specific Funding in Fiscal Year 2006. | | | | |
| --- | --- | --- | --- | --- |
| **Predictor** | **Predicted change in funding associated with a 2-fold**  **increase in the predictor*** | | | |
|  | **Relative Increase** | **95% CI** | ***P*-Value** | **Adjusted**  **R-squared Value** |
| **Disability-Adjusted Life-Years** | | | | |
| North America |  |  |  |  |
| 2004 | 1.37 | (1.16-1.63) | 0.001 | 0.33 |
| 2015^†^ | 1.27 | (1.04-1.56) | 0.02 | 0.16 |
| 2030^†^ | 1.25 | (1.03-1.51) | 0.02 | 0.15 |
| World |  |  |  |  |
| 2004 | 1.37 | (1.14-1.63) | 0.001 | 0.30 |
| 2015 | 1.35 | (1.13-1.62) | 0.002 | 0.29 |
| 2030 | 1.36 | (1.14-1.63) | 0.002 | 0.31 |

* Predictors and outcome are log-transformed to reduce positive skew.

^†^ Data pertains to world market economies as defined by the World Health Organization.
